# Steroid pulse therapy for acute T-cell-mediated rejection after kidney transplantation: mechanisms, evidence, and unresolved questions

**DOI:** 10.3389/fimmu.2026.1867698

**Published:** 2026-06-12

**Authors:** Sami Siam, Göran Ramin Boeckel, Chalid Hasan, Hermann Pavenstädt, Klemens Budde, Stefan Reuter

**Affiliations:** 1 Department of Medicine D, Transplant Nephrology, University Hospital Münster, Münster, Germany; 2 Department of Nephrology and Medical Intensive Care, Charité Universitätsmedizin Berlin, Berlin, Germany

**Keywords:** acute rejection, glucocorticoids, immunometabolism, kidney transplantation, methylprednisolone, non-genomic mechanisms, pulse therapy, T-cell-mediated rejection

## Abstract

Intravenous methylprednisolone (MP) pulse therapy remains the standard first-line treatment for acute T-cell-mediated rejection following kidney transplantation. However, the dose and duration of this therapy were established empirically more than three decades ago rather than by contemporary rigorous dose-finding trials. The most common treatment regimens consist of 250–500 mg MP daily for three to five days, but substantial between-center variability persists. This narrative review explores the historical development of steroid pulse dosing, the available comparative clinical data, and the biological rationale underlying current practice. The commonly used dose range of 250–500 mg daily for three to five days rests largely on historical convention rather than on contemporary dose-finding evidence. A series of small prospective and randomized studies failed to demonstrate superior efficacy of more intensive regimens when compared with doses broadly resembling current practice. Mechanistically, glucocorticoids act through genomic and rapid non-genomic pathways, supporting a hypothesis-generating dose-window concept rather than a simple linear dose-response model. Contemporary data further indicate that clinical response does not reliably predict histologic resolution and that MP pulse rejection treatment carries significant toxicity. Overall, steroid pulse therapy remains biologically plausible and clinically entrenched; however, the optimal dose and duration have yet to be established under current tacrolimus- and mycophenolate-based immunosuppression. More precise response monitoring will also be required to develop more effective and less toxic treatment regimens.

## Introduction

Steroid pulse therapy (SPT) occupies a distinctive place in kidney transplantation. It is widely used for acute T-cell-mediated rejection (TCMR), yet its dose and duration remain poorly defined. Across transplant centers, intravenous methylprednisolone (MP) is commonly prescribed at doses of 250–500 mg daily for three to five days, but no modern randomized dose-finding trial has established this range as superior to lower or higher alternatives under contemporary tacrolimus/mycophenolate-based immunosuppression (1–4). What appears standardized is therefore largely historical convergence.

Despite major reductions in acute rejection rates over recent decades with contemporary immunosuppressive regimens, TCMR remains a clinically important cause of allograft dysfunction and inferior graft outcomes (1, 5, 6). Clinically apparent TCMR occurs in approximately 8–15% of kidney transplant recipients, mainly within the first year after transplantation, although reported rates depend strongly on biopsy strategy, immunological risk, and case mix (1, 2, 7, 8). Cohorts including surveillance biopsies may detect additional subclinical or borderline inflammatory lesions, whereas recent standard-risk protocol-biopsy data suggest substantially lower rates in uneventful recipients (8). In the Harmony trial, TCMR occurred in approximately 8% of patients, Banff grade >I TCMR in about 3%, and steroid-resistant rejection in about 2% (7). Importantly, graft-loss adjudication studies show that TCMR remains a relevant contributor to death-censored graft failure (6). Reversal rates for first episodes are commonly reported around 60–75%, with a substantial minority proving steroid-resistant and requiring escalation to T-cell-depleting antibody therapy (1, 9, 10).

This evidence gap is clinically relevant for three reasons. First, SPT is frequently used, yet substantial practice variation persists across Europe, Canada, and the United States (3, 4, 11, 12). Second, rejection therapy contributes to infectious and metabolic complications, and recent data suggest that infection after rejection treatment may be a major driver of inferior patient and graft survival (13). Third, the field has changed profoundly since the original steroid studies were conducted: contemporary recipients are managed with tacrolimus/mycophenolate-based maintenance regimens, Banff-defined histology, donor-specific antibody assessment, and, in selected centers, surveillance or follow-up biopsies (1, 3, 5, 11, 12). A dose that became customary before these diagnostic and therapeutic standards should not be mistaken for one validated in the current era.

This review addresses four linked questions: how high-dose SPT became established in kidney transplantation, whether comparative clinical evidence supports one pulse dose over another, whether a biological rationale exists for the commonly used 250–500 mg MP range, and how glucocorticoids exert anti-rejection effects beyond the traditional shorthand of general anti-inflammatory action. Several recent publications have highlighted practice heterogeneity, limited trial evidence, and the poor correlation between functional and histologic response after TCMR treatment. The specific contribution of this review is to integrate four evidence layers that have usually been considered separately: historical steroid dose-comparison studies, contemporary practice and response-monitoring data, modern glucocorticoid receptor biology including non-genomic and immunometabolic effects, and glucocorticoid-stewardship considerations. This synthesis frames SPT not simply as an inherited anti-rejection intervention, but as an unresolved dose-development and response-monitoring problem in contemporary kidney transplantation.

## Historical origins of steroid pulse therapy

Pulse steroid therapy entered transplant practice early. In 1972, Feduska and colleagues reported reversal of renal allograft rejection with intravenous methylprednisolone pulse therapy, establishing the concept that brief exposure to very high glucocorticoid concentrations could reverse rejection without prolonged escalation of baseline steroid exposure (14). This approach was conceptually attractive in the early transplant era, when maintenance immunosuppression was limited and clinicians needed a rapid, reversible anti-inflammatory intervention.

The following decade shaped practice with several small studies, but not through formal dose optimization. Kauffman and colleagues evaluated high-dose bolus MP perioperatively in kidney transplantation but did not demonstrate any prophylactic benefit (15). More importantly, a randomized trial treated patients with established renal allograft rejection with very high-dose (30 mg/kg) or lower-dose (3 mg/kg) of corticosteroids found no clear therapeutic advantage of the higher-dose regimen (16, 17). Their data suggested, if anything, a signal toward more infectious complications and septic deaths with the higher dose (16, 17). Shortly thereafter, Gray and colleagues compared high-dose oral prednisolone with intravenous MP for treating rejection and found broadly comparable reversal rates (~60%), further arguing against a simple linear dose-intensity model (18).

By current standards, these early studies are methodologically limited. They predate the Banff classification of TCMR, calcineurin inhibitor-based maintenance immunosuppression, donor-specific antibody testing, and contemporary definitions of treatment success. Sample sizes were small, and histologic endpoints were not standardized (16–22). Nevertheless, the historical message is strikingly consistent: escalation to substantially higher steroid exposure did not improve rejection reversal. The lowest clearly effective intravenous regimens in these studies were approximately 3 mg/kg/day MP for three to five days or fixed-dose 250 mg/day for four days, whereas escalation to 15–30 mg/kg/day or 1, 000 mg/day did not show superior efficacy (16–22). Thus, the historical data support short multi-day pulses, but do not define a precise minimum effective dose.

The same pattern persisted during the 1980s and early 1990s. In children, Orta-Sibu et al. compared high-dose intravenous MP at 600 mg/m²/day for three days with oral prednisolone at 3 mg/kg/day for three days and found no superiority of the intravenous high-dose regimen in reversing acute rejection (19). Park et al. compared 250 mg with 1, 000 mg MP daily for four days and found no significant difference in response (20). Lui et al. reported similar efficacy with 3 mg/kg/day and 15 mg/kg/day MP for three days in cyclosporine-treated recipients (21). De Backer et al. compared 8 mg/kg every second day for three doses with 3 mg/kg/day for five days after OKT3 prophylaxis and again found very similar rejection reversal rates (22). Across these small prospective datasets, short multi-day steroid pulses appeared effective, but escalation to substantially higher steroid exposure consistently failed to demonstrate convincing superiority (17–22).

The current common practice of 250–500 mg methylprednisolone daily for approximately three days therefore appears to have emerged from historical experience and repeated description in later reviews, guidelines, and practice surveys rather than from a systematic dose-development program (1–4, 11, 12, 23). It became a de facto standard, although the precise dose and duration remained empirical.

## Current practice and the landscape of heterogeneity

The KDIGO clinical practice guideline for kidney transplant recipients recommends corticosteroids as initial therapy for acute cellular rejection and notes that the dose and duration of treatment have not been well defined by randomized trials; it further describes intravenous MP 250–500 mg daily for three days as common practice (23). In contemporary practice, many centers still use 250–500 mg MP daily for three to five days, a convention shaped more by expert opinion and historical precedent than by direct comparative evidence (1, 2, 4).

Cooper et al. described pulse MP, typically 250–500 mg daily for three to five days, as the conventional initial treatment for Banff borderline and Banff grade I rejection. Escalation to rabbit antithymocyte globulin (ATG) is usually reserved for steroid-resistant disease or more severe Banff grade II/III lesions (**Supplementary Table S4**) (1). A recent European practice survey confirmed that most centers use corticosteroids as primary therapy for borderline rejection and Banff grade IA–IIB TCMR, whereas ATG is mainly reserved for second-line treatment or more severe rejection (3). Canadian practice patterns appear broadly aligned with this steroid-first approach, while the US survey suggests earlier and more frequent use of lymphocyte-depleting therapy in higher-grade TCMR (11, 12).

The European, Canadian, and US surveys also revealed that heterogeneity extends beyond treatment choice. Protocol or surveillance biopsies are not consistently performed, definitions of protocol biopsy vary, and biopsy-based molecular diagnostics are not routinely incorporated into clinical practice (3, 11, 12). Budde succinctly highlighted the core problem: high-quality evidence supporting current anti-rejection regimens remains sparse, and much of current practice is inherited from the early days of transplantation (4). He further emphasized the need for standardized definitions of treatment response and corticosteroid refractoriness, as well as more precise recommendations for anti-TCMR therapy and follow-up (4).

Thus, the current guidance is clinically coherent but rests on limited and heterogeneous evidence. Steroid pulses remain standard because they are longstanding, widely available, inexpensive, rapidly acting, and biologically plausible, not because the optimal dosage has been proven (1–4, 23). The main dimensions of this practice heterogeneity are summarized in **Supplementary Table S1**.

## Mechanistic rationale: established, extrapolated, and hypothesis-generating evidence

**Figure 1:**
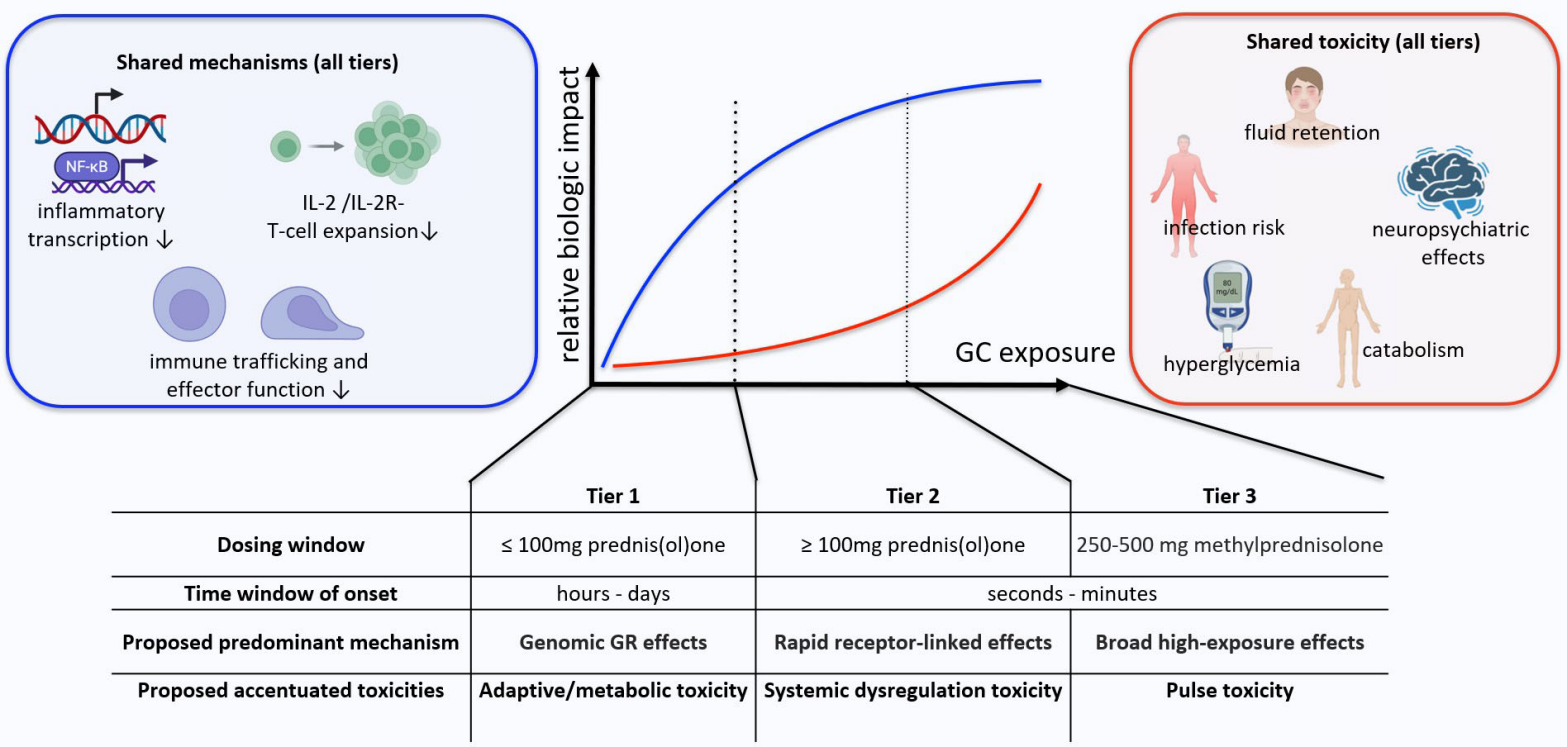
Hypothetical dose-window model for steroid pulse therapy in acute TCMR. The figure integrates the standardized glucocorticoid dose nomenclature proposed by Buttgereit et al. with a hypothetical tier-dependent framework of glucocorticoid action in acute TCMR (35). Conventional glucocorticoid exposure below approximately 100 mg prednisone equivalent is presumed to be dominated by genomic glucocorticoid receptor (GR)-mediated effects, whereas very high and pulse-dose exposure may increasingly recruit rapid non-genomic, membrane-associated, and broader systemic mechanisms. Steroid pulse therapy is conventionally defined as >250 mg prednisone equivalent daily for one or a few days. Commonly used transplant regimens of 250–500 mg methylprednisolone correspond to approximately 312–625 mg prednisone equivalent and therefore fall within the pulse-dose range. The proposed saturation concept is hypothetical and suggests that escalating glucocorticoid exposure may progressively increase toxicity while therapeutic benefit approaches a plateau. This model should not be interpreted as evidence that 250 mg and 500 mg methylprednisolone are clinically equivalent. The model further proposes overlapping biologic effects and toxicities across tiers (blue and red boxes, respectively), while higher exposure tiers may be characterized by predominant mechanistic and toxicity patterns. Proposed predominant mechanisms include genomic GR-mediated transrepression/transactivation and cytokine suppression (Tier 1), rapid signaling interference with reduced IL-2/IL-2R/JAK-STAT signaling (Tier 2), and broader membrane-associated, immunometabolic, and potential GR desensitization/downregulation effects at pulse exposure (Tier 3). Shared toxicities may vary in severity and clinical phenotype across exposure tiers and between individual patients depending on susceptibility, comorbidities, and pre-existing risk factors. For example, neuropsychiatric manifestations may range from mood changes to emotional lability or psychosis, whereas cardiovascular effects may range from hypertension to hypertensive crises. GC, glucocorticoid; GR, glucocorticoid receptor; IL-2R, interleukin-2 receptor; MP, methylprednisolone; NF-κB, nuclear factor kappa-light-chain-enhancer of activated B cells; TCMR, T-cell-mediated rejection. Created with BioRender.com.

The mechanistic evidence discussed below should be interpreted according to its level of directness. Suppression of inflammatory transcription, lymphocyte activation, and IL-2-dependent signaling is biologically relevant to TCMR, but much of the detailed glucocorticoid receptor and immunometabolic literature derives from experimental immunology, rheumatology, or non-transplant models rather than from steroid-treated human kidney allograft TCMR. We therefore distinguish established transplant-relevant principles from extrapolated mechanisms and hypothesis-generating concepts. This hierarchy is central to the dose-window concept shown in **Figure 1**, which is intended as a conceptual framework rather than a validated dosing model.

### Genomic pathways: transrepression, transactivation, and immune deviation

Glucocorticoids exert their canonical immunosuppressive effects through the cytosolic glucocorticoid receptor (GR), a ligand-activated transcription factor (24, 25). After ligand binding, the receptor complex translocates to the nucleus and modulates gene expression through transrepression and transactivation. Through transrepression, activated GR physically interacts with nuclear factor kappa-light-chain-enhancer of activated B cells (NF-κB) and activator protein-1 (AP-1), thereby blocking transcription of pro-inflammatory cytokines, including interleukin (IL)-2, IL-6, tumor necrosis factor α (TNF-α), and interferon γ (IFN-γ). Auphan et al. demonstrated that glucocorticoids inhibit NF-κB activity by inducing IκB-α synthesis, providing a molecular explanation for broad suppression of pro-inflammatory cytokine production (26). Through transactivation, GR induces anti-inflammatory mediators, including glucocorticoid-induced leucine zipper (GILZ), DUSP1/MKP-1, and annexin-1. However, contemporary GR biology is more complex than the old binary of “transactivation equals side effects” and “transrepression equals benefit”, involving multiple isoforms, ligand-specific conformations, context-dependent co-regulators, and cell-specific transcriptional outputs (25, 27, 28).

Glucocorticoids also selectively suppress Th1 cytokines (e.g., IL-12 and IFN-γ) while relatively preserving Th2 responses (e.g., IL-4 and IL-10). This induces an immunological shift that is particularly relevant in TCMR, which is predominantly driven by Th1 and cytotoxic T-cell responses (29). High-dose glucocorticoids further induce apoptosis in activated T lymphocytes. CD4+ T cells are more susceptible than CD8+ T cells, with regulatory T cells (Tregs) showing relative resistance. This may contribute to the restoration of immune homeostasis following pulse therapy (30, 31).

### Non-genomic pathways and the hypothetical dose-window concept

Beyond the classical genomic pathway (onset: hours to days), glucocorticoids exert rapid non-genomic effects (onset: seconds to minutes), which become particularly relevant at high and suprapharmacological exposures. Buttgereit et al. described three tiers of glucocorticoid action: 1) genomic effects via the cytosolic GR at low to medium doses, 2) rapid non-genomic effects associated with receptor-linked signaling mechanisms at higher doses, and 3) non-specific membrane effects at very high exposures, including altered ion transport and mitochondrial function (32–35). Cytosolic GR-mediated genomic effects are generally considered dose-limited, and very high glucocorticoid exposure may increasingly recruit rapid non-genomic mechanisms. However, available data do not define a transplant-specific saturation point or a biologically validated MP dose window for acute kidney allograft TCMR. Pediatric autoimmune-disease data showed marked lymphocyte GR downregulation after intravenous pulse therapy with 10–15 mg/kg/day prednisolone-equivalent exposure, and adult rheumatologic data support dose-dependent GR downregulation; however, these studies were primarily based on radioligand binding assays and did not directly assess downstream signaling, receptor conformational state, or transcriptional activity (36, 37). Thus, while these data are consistent with the concept that very high steroid doses may shift the balance toward additional non-genomic mechanisms, they do not establish a strict mechanistic threshold. In the transplant setting, the commonly used 250–500 mg MP range should therefore be regarded as a pragmatic high-exposure regimen, not as a biologically proven optimal range or mechanistic ceiling. The available clinical literature does not show convincing benefit from escalation above this range, but it also does not prove equivalence between 250 mg and 500 mg MP (1, 2, 4, 20–22).

### The IL-2/IL-2R/JAK-STAT axis: a key target in TCMR

A mechanistic layer of particular relevance to TCMR has emerged from studies of IL-2 signaling. Glucocorticoids inhibit IL-2-induced JAK-STAT signaling in primary T cells, including STAT5 activation and IL-2 receptor/JAK3 expression (38). More recent work in human primary T cells suggests that suppression of the IL-2/IL-2R axis is a major contributor to the immunosuppressive effect of glucocorticoids, whereas proximal T-cell receptor signaling is less affected (39). Together, these findings provide a biologically plausible explanation for why steroid pulses can rapidly blunt activated alloreactive T-cell responses.

### Immunometabolic reprogramming

A growing body of evidence suggests that cellular metabolism is a relevant target of glucocorticoid action. Experimental studies indicate that glucocorticoids can suppress pro-inflammatory glycolytic programs and promote anti-inflammatory metabolic reprogramming in immune cells (40–42). These findings support the broader concept that SPT may constrain immune effector function not only transcriptionally but also metabolically. However, direct evidence in alloreactive T cells during human kidney allograft rejection remains limited. Macrophages are a recognized component of the inflammatory infiltrate in acute TCMR and may shape the local rejection microenvironment, but macrophage-based glucocorticoid studies remain indirect for this specific indication. Accordingly, immunometabolic reprogramming should currently be viewed as a plausible, hypothesis-generating mechanism rather than direct evidence for steroid responsiveness in human kidney allograft TCMR.

## Cross-organ evidence: indirect support only

Evidence from other solid-organ transplant settings provides indirect support for a non-linear dose-response relationship, but should not be overinterpreted for kidney allograft TCMR. In heart transplantation, reduced-intensity steroid strategies have been reported as feasible in clinically stable rejection episodes, and Kobashigawa et al. directly compared 1, 000 mg intravenous MP daily for three days with oral prednisone 100 mg daily for three days followed by tapering to the previous maintenance dose over 14 days. Rejection resolution at four weeks was similar in both groups, arguing against a mandatory requirement for very high intravenous peak exposure in asymptomatic moderate cardiac rejection (43, 44). In liver transplantation, Volpin et al. compared 1, 000 mg intravenous MP once followed by a six-day taper from 200 to 20 mg/day with 1, 000 mg intravenous MP daily for three consecutive days. The tapered schedule achieved higher histological resolution and fewer infections than three consecutive 1, 000 mg pulses (45). These cross-organ studies do not define an optimal MP dose for acute kidney allograft TCMR, but they reinforce the broader principle that maximal peak exposure is not consistently associated with superior rejection reversal across solid-organ transplant settings.

Taken together, these pathways suggest that SPT does not act through a single dominant mechanism, but through coordinated interruption of inflammatory transcription, T-cell proliferation signals, leukocyte trafficking, and cellular bioenergetics. The mechanistic layers relevant to SPT are summarized in **Supplementary Table S2**. This framework provides a coherent biological rationale for brief high-exposure steroid treatment, but it remains insufficient to define a validated optimal MP dose or to predict which patients benefit from escalation within the 250–500 mg range.

## Comparative pharmacology and rationale for methylprednisolone

Given the mechanistic rationale for pulse dosing, the choice of glucocorticoid preparation becomes a practical consideration. MP is the standard agent for pulse therapy because it has several pharmacological advantages: a 5-fold higher glucocorticoid potency than hydrocortisone, low mineralocorticoid activity, excellent intravenous bioavailability, and an intermediate biological half-life (12–36 hours), which provides sustained, yet not excessively prolonged, immunosuppression (46, 47). Despite its 25- to 30-fold glucocorticoid potency and virtual absence of mineralocorticoid activity, dexamethasone is generally not used for pulse therapy in transplantation due to its prolonged biological half-life (36–72 hours), which may increase the risk of sustained adrenal suppression and metabolic complications. The pharmacological properties of clinically relevant glucocorticoids are summarized in **Supplementary Table S3**.

## Clinical evidence in the contemporary era

### Historical dose-comparison studies

The updated 2017 Cochrane systematic review by Webster et al., included 31 randomized controlled trials with 1, 680 patients and demonstrated that lymphocyte-depleting therapy (ATG) is probably superior to SPT for reversing TCMR (RR 0.50; 95% CI 0.30–0.82), though it resulted in significantly more adverse events (10). However, most of the included trials predated modern immunosuppressive regimens and used heterogeneous steroid and ATG-dosing protocols.

The key comparative studies are summarized in **Table 1**. Across several small prospective datasets from the 1970s through the early 1990s, SPT appeared to be an effective intervention; however, escalation to higher steroid exposure consistently failed to demonstrate convincing superiority (16–22). The major weakness of the literature is not the absence of efficacy signals, but the absence of contemporary dose discrimination. Thus, the unresolved question is not whether corticosteroids can reverse TCMR, but how dose, duration, tapering, and response should be defined under contemporary tacrolimus/mycophenolate-based immunosuppression. Notably, the oral prednisone taper that typically follows the IV pulse has received even less scrutiny. Its duration, starting dose, and tapering speed are empirical and vary widely across centers. The cumulative glucocorticoid exposure from the taper phase may equal or exceed that of the pulse itself (2, 9). The ongoing TACKLE-IT trial (NCT06474273) is therefore an important randomized effort to address both MP pulse dose and oral steroid taper under contemporary immunosuppression.

**Table 1:** Key comparative studies informing steroid pulse dosing in kidney allograft rejection.

Study	Era/population	Compared regimens	Main finding	Key limitation
Feduska et al., 1972 (14)	Early renal Tx	IV MP pulse therapy	Established feasibility of pulse therapy for rejection reversal	No comparator or dose optimization
Stromstad/Kauffman 1978/79 (16, 17)	Kidney allograft rejection	30 mg/kg vs 3 mg/kg corticosteroid regimen	No advantage of higher dose; more infections	Pre-Banff, small, older maintenance
Gray et al., 1978 (18)	Renal allograft rejection	High-dose oral pred vs IV MP	Similar reversal rates (~60%)	Pre-modern era
Orta-Sibu et al., 1982 (19)	Pediatric kidney transplantation	IV MP 600 mg/m²/day x 3d vs oral pred 3 mg/kg/day x 3d	No advantage of IV high-dose	Small pediatric study
Park et al., 1984 (20)	Acute rejection	250 mg vs 1, 000 mg daily x 4d	No significant difference	Small sample size
Lui et al., 1989 (21)	CsA-treated recipients	3 mg/kg/d vs 15 mg/kg/d x 3d	Similar efficacy	Small; older background IS
De Backer et al., 1992 (22)	After OKT3 prophylaxis	8 mg/kg q2d x 3 vs 3 mg/kg/d x 5	Similar reversal rates	Specialized population
Shinn et al., 1999 (48)	Acute rejection	500 mg/d x 3 (response kinetics)	Response unreliable before day 5	No dose comparison

Tx, transplantation; MP, methylprednisolone; pred, prednisolone; CsA, cyclosporine; IS, immunosuppression.

### Treatment response assessment

Treatment success is often assessed imperfectly. Shinn et al. demonstrated that responders and nonresponders to pulse MP could not be reliably distinguished early after treatment, with serum creatinine separating the groups only around day 5 (48). A systematic review and meta-analysis by Ho et al. revealed substantial variation in treatment approaches for borderline and Banff grade IA/IB TCMR under tacrolimus/mycophenolate maintenance therapy, with persistent or recurrent rejection remaining prevalent and histologic remission assessed in only a minority of studies (49). Aziz et al. further demonstrated that kidney function response correlates poorly with histologic response: complete histologic response rates were relatively favorable for borderline and grade IA lesions but substantially lower for grade IB and IIA lesions, and creatinine-based improvement did not reliably reflect tissue-level resolution (50). Similarly, Rampersad et al. showed that persistent or subsequent TCMR on follow-up biopsies is common and clinically meaningful, with adverse consequences for graft survival (5). Molecular analyses further suggest that some kidney transplant recipients may harbor subthreshold rejection activity not captured by conventional histology, including lesions classified as no rejection by current criteria (51). Direct evidence for transcriptomic monitoring after steroid-treated TCMR remains limited; however, analogous work in antibody-mediated rejection illustrates how repeated molecular phenotyping can reveal response dynamics beyond routine histology or kidney function alone (52, 53).

Budde highlights another challenge: contemporary biopsies often no longer reveal isolated “classical” TCMR. Transplant physicians are increasingly confronted with mixed pathologies, particularly in marginal kidneys with delayed or slow graft function. In these cases, tubulointerstitial inflammation may coexist with acute tubular injury, capillaritis, or chronic structural lesions. In this setting, decisions about administering SPT and evaluating treatment response become considerably more difficult. Further, this problem is not only methodological but also definitional. As emphasized, the lack of standardized definitions for treatment response and steroid-refractory rejection in current practice makes inter-center comparisons difficult and complicates the interpretation of retrospective data and the design of prospective trials (4).

The clinical implication is that creatinine kinetics alone are insufficient to define treatment success. When clinical stakes are high, follow-up biopsy is more informative than short-term functional improvement for determining whether inflammation has resolved (5, 50). Biopsy-based molecular diagnostics may further refine response assessment by helping distinguish persistent alloimmune activity from morphologically ambiguous or residual inflammatory lesions (51, 54, 55). At present, however, these tools should be regarded as complementary rather than replacement diagnostics, as their interventional utility for selecting steroid dose or defining steroid failure has not yet been established. Consequently, in the ongoing TACKLE-IT trial, treatment success is assessed at 12 weeks using a composite endpoint including histologic resolution, improvement in allograft function, and avoidance of rescue therapy.

### Steroid resistance: mechanisms and clinical predictors

Approximately 25–30% of acute TCMR episodes do not respond to SPT. In a comprehensive analysis of steroid-resistance mechanisms, GR gene polymorphisms, increased P-glycoprotein (P-gp) expression enhancing cellular glucocorticoid efflux, and the degree of the inflammatory milieu were identified as key determinants (56). It remains unclear whether dose escalation can meaningfully overcome resistance mechanisms such as P-gp-mediated efflux, or whether TCMR-associated immune activation may become steroid-insensitive regardless of dose. Awadain et al. identified low calcineurin inhibitor trough levels at the time of rejection as an important clinical risk factor for steroid resistance (57). Older studies showing successful conversion from cyclosporine to tacrolimus in refractory rejection further support the concept that adequate concomitant maintenance immunosuppression, including CNI exposure and antimetabolite dosing, is central to successful TCMR treatment (58–60). In steroid-resistant rejection, the established approach remains escalation to lymphocyte-depleting therapy (23, 61). The relationship between steroid dose and resistance mechanisms is an important knowledge gap: if resistance is mediated by receptor polymorphisms, efflux pumps, or insufficient background immunosuppression, increasing the steroid dose may not be the answer.

## Borderline rejection: the hardest test case

Borderline changes are the most controversial area regarding SPT. Formally, the Banff classification does not define borderline changes as definitive rejection, but as a separate category suspicious for acute TCMR (62). In routine practice, however, many centers still treat borderline changes, especially when detected on indication biopsy (1, 3). The evidence for this approach is mixed and highly context-dependent, and the diagnosis ultimately depends on histologic assessment with limited interobserver reproducibility and potential sampling error (63, 64).

This uncertainty is likely both clinical and biological. De Freitas et al. demonstrated that biopsies classified as “borderline rejection” are not a uniform entity but rather a heterogeneous group positioned in a continuum of rejection activity between non-rejection and definite TCMR (65). Borderline biopsies shared some inflammatory features with TCMR while lacking the overall inflammatory burden of unequivocal rejection. This finding supports the idea that the borderline category encompasses biologically diverse processes rather than a single treatment-responsive lesion. Borderline inflammation should therefore be interpreted in the context of biopsy indication, inflammatory burden, and graft dysfunction rather than automatically triggering treatment.

For minimal inflammatory lesions, especially i0 borderline infiltrates, the data do not support routine steroid treatment. Dale et al. found no improvement in renal function, follow-up histology, rejection-free survival, graft survival, or patient survival in recipients with i0 borderline infiltrates with corticosteroid treatment (66). More recently, Kirn et al. evaluated SPT for borderline TCMR detected on protocol biopsy and observed no clear increase in histologic resolution. However, they found that treated patients showed more interstitial fibrosis/tubular atrophy progression and a less favorable eGFR trajectory (67).

Conversely, Palmisano et al. reported preliminary evidence indicating that treatment may be beneficial when borderline TCMR is identified through a clinically indicated biopsy rather than surveillance biopsy (68). This distinction is plausible because borderline inflammation in a dysfunctional graft may reflect a biologically different state from low-grade inflammatory lesions detected on surveillance biopsy in otherwise stable recipients.

For SPT, this distinction is crucial because borderline lesions are the setting in which overtreatment is most likely and treatment responsiveness is most context-dependent. If the evidence for benefit is weak or context-restricted, dose minimization becomes even more important.

## Toxicity and stewardship

The historical debate surrounding the efficacy of steroid pulse therapy has often overlooked toxicity. Modern data increasingly depict rejection treatment as a trade-off between immune control and collateral harm. This mirrors the broader glucocorticoid-stewardship principle established in inflammatory medicine: glucocorticoids should be used at the lowest effective dose and for the shortest duration compatible with disease control (69). Gupta et al. demonstrated that treatment for allograft rejection is associated with a significantly higher cumulative incidence of subsequent infection (13). Notably, in that analysis, infection—rather than rejection itself—was associated with inferior patient and graft survival.

This does not argue against treating genuine rejection. Rather, it argues against therapeutic complacency. If higher steroid doses have not convincingly demonstrated superior efficacy, then additional exposure should be justified by a plausible expectation of incremental benefit (13, 16–22). The same logic applies to repeated pulses for histologically ambiguous lesions, especially when the biopsy phenotype and clinical context suggest a low likelihood of meaningful benefit (66–68).

A stewardship approach to steroid pulses should include accurate biopsy-based diagnosis, dose discipline rather than ritual escalation, reassessment of histologic response in selected patients, and active prevention and monitoring of infectious complications. Steroid toxicity should be considered as both immediate and cumulative. Acute pulse-related adverse effects include hyperglycemia, fluid retention, hypertension, neuropsychiatric symptoms, oral candidiasis, gastrointestinal irritation or bleeding, and increased susceptibility to infection (13, 70, 71). With repeated pulses or prolonged oral tapers, cumulative glucocorticoid exposure may also contribute to post-transplant diabetes, weight gain, skin and cosmetic changes, bone loss, fractures, and avascular necrosis; in kidney transplant recipients, early corticosteroid withdrawal has been associated with reduced fracture risk (9, 72). Toxicity is not solely a function of cumulative exposure. For selected complications, particularly osteonecrosis, the highest daily dose, parenteral pulse exposure, and the duration of very high-dose exposure may also contribute to risk, even though a universally safe threshold cannot be defined (73). Preventive measures should therefore be risk-adapted and include glucose and blood-pressure monitoring, infection and fungal surveillance, individualized gastric protection in patients at increased gastrointestinal risk, and bone-health measures aiming to mitigate bone loss, including vitamin D assessment and management of chronic kidney disease–mineral and bone disorder (CKD-MBD) or secondary hyperparathyroidism when repeated or prolonged steroid exposure is used (23, 74).

## Practical implications for current practice

Several practical conclusions emerge from the reviewed evidence. First, SPT remains a reasonable first-line treatment for Banff grade I TCMR and for selected borderline lesions diagnosed by indication biopsy. First-line treatment for more severe Banff II/III lesions is more controversial and differs between regions: US centers appear to use lymphocyte-depleting therapy earlier and more frequently, whereas European and Canadian practice more often begins with MP pulse therapy (1–3, 11, 12).

Second, comparative evidence does not establish a consistent clinical superiority of 500 mg over 250 mg methylprednisolone, although a mechanistic incremental effect of the higher dose cannot be excluded. Historical literature suggests that lower pulse doses may often be sufficient, but does not define which patients, if any, benefit from escalation within the 250–500 mg range (17–22).

Third, steroids do not act alone in reversing rejection. Adequate concomitant immunosuppression, especially sufficient calcineurin inhibitor exposure and appropriate antimetabolite dosing, is crucial for successful treatment (57–60).

Fourth, histology matters more than creatinine alone. When the clinical stakes are high, follow-up biopsy is more informative than creatinine kinetics alone for determining whether inflammation has actually resolved (5, 50).

Fifth, not all borderline lesions should be treated identically. Borderline lesions diagnosed by indication biopsy are commonly treated with steroid pulses, whereas treatment of borderline lesions in protocol biopsies is more controversial, and minimal i0 lesions appear to be poor candidates for automatic pulse therapy (66–68).

Sixth, the oral prednisone taper following pulse therapy remains an equally unresolved question. The duration and intensity of the taper are entirely empirical, and cumulative glucocorticoid exposure from the taper may substantially contribute to metabolic and infectious complications. This component of rejection treatment has received even less attention than the IV pulse dose itself.

## Evidence gaps and the TACKLE-IT trial

**Table 2** summarizes critical evidence gaps in SPT for TCMR. The TACKLE-IT trial (NCT06474273) is the first prospective randomized study to address longstanding uncertainties regarding the dose of intravenous methylprednisolone and the tapering of oral prednisone in acute TCMR. Future progress will require dose comparison, standardized response definitions, and better phenotyping of treated lesions. Non-invasive biomarkers such as donor-derived cell-free DNA and tissue-based molecular diagnostics may improve treatment stratification and follow-up monitoring, but neither currently defines a validated steroid algorithm for acute TCMR. Even well-designed prospective trials must therefore contend with substantial biological and diagnostic heterogeneity across the rejection continuum.

**Table 2:** Critical evidence gaps in glucocorticoid pulse therapy for TCMR.

Evidence cap	Clinical relevance	Status	Ref.
Optimal IV MP dose (high vs low)	Dose minimization could reduce adverse effects	TACKLE-IT (NCT06474273)	(10, 16–22)
Optimal oral prednisone taper	Duration and intensity entirely empirical	TACKLE-IT (NCT06474273)	(2, 9)
Steroid pulse under modern IS (TAC/MMF)	No RCT under current standard of care	Unaddressed	(1–4)
Biomarker- and molecular pathology-guided treatment	May improve phenotyping, response assessment, and risk stratification beyond creatinine alone	Research phase (dd-cfDNA, biopsy transcriptomics/MMDx, TCR)	(51, 54, 55)
Role in subclinical/borderline TCMR	Questionable benefit; risk of overtreatment	Retrospective data only	(65–67)
Steroid resistance and dose relationship	Whether higher doses overcome resistance mechanisms	Unanswered	(56, 57)
Molecular resolution of TCMR after therapy	Histology and kidney function may underestimate persistent alloimmune activity	Exploratory/not standardized	(1, 51, 54, 55)

MP, methylprednisolone; IS, immunosuppression; TAC, tacrolimus; MMF, mycophenolate mofetil; GC, glucocorticoid; dd-cfDNA, donor-derived cell-free DNA; TCR, T-cell receptor.

## Conclusions

Although SPT for acute TCMR after kidney transplantation is biologically plausible, historically entrenched, and clinically useful, its dosing remains poorly evidence-based. The biological rationale for pulse dosing has become more coherent in recent years, particularly through better understanding of non-genomic signaling, IL-2/IL-2R/JAK-STAT suppression, and immunometabolic reprogramming. However, several mechanistic elements remain extrapolated from non-transplant or non-TCMR settings, and the historical prospective literature does not demonstrate consistent superiority of higher over lower-dose regimens. Furthermore, modern studies show that short-term kidney function response is an unreliable surrogate for histologic resolution.

After more than 50 years of use, we still cannot answer the fundamental clinical question: What is the minimum effective methylprednisolone dose for treating acute TCMR under contemporary immunosuppression? Until the results of TACKLE-IT and similar trials are available, clinicians must acknowledge that current practice is guided more by tradition than by evidence. In the meantime, an approach combining accurate diagnosis, dose discipline, histologic reassessment, and infection surveillance remains essential for informed clinical decision-making.

## Supplementary material

Supplementary Table S1 Practice heterogeneity in glucocorticoid pulse therapy for TCMR.

Supplementary Table S2 Mechanistic layers of glucocorticoid action relevant to steroid pulse therapy in TCMR.

Supplementary Table S3 Comparative pharmacological properties of clinically used glucocorticoids.
