# Supplementary material for: Steroid pulse therapy for acute T-cell-mediated rejection after kidney transplantation: mechanisms, evidence, and unresolved questions
Source: Front Immunol. 2026 Jun 12;17:1867698. doi: 10.3389/fimmu.2026.1867698 (PMC13303207; doi:10.3389/fimmu.2026.1867698)
Supplement: Supplementary Table S1 — Practice heterogeneity in glucocorticoid pulse therapy for TCMR. [file Supplementaryfile1.docx]

Supplemental

Search strategy

This narrative review was informed by a scoping literature search rather than formal systematic-review procedures. A structured PubMed/MEDLINE search was performed for studies on steroid pulse therapy in kidney allograft rejection from January 1, 1970, to March 27, 2026. The search combined Medical Subject Headings and free-text terms related to kidney transplantation (“kidney transplantation” OR “renal transplantation” OR “renal allograft”), corticosteroids (“methylprednisolone” OR “glucocorticoid” OR “steroid pulse” OR “corticosteroid”), acute rejection (“acute rejection” OR “T-cell-mediated rejection” OR “cellular rejection”), and study design or dose (“dose” OR “dose-response” OR “randomized” OR “comparative”). Additional focused searches addressed follow-up-biopsy studies after rejection treatment, glucocorticoid mechanisms of action, cross-organ steroid dose-comparison data, steroid resistance mechanisms, and steroid toxicity. ClinicalTrials.gov was searched for ongoing or completed dose- or taper-comparison trials in kidney transplant rejection (search date: March 2026). Reference lists of key articles and reviews were hand-searched for additional relevant publications. Embase, Scopus, Web of Science, the Cochrane Library beyond the cited Cochrane review, WHO ICTRP, conference abstracts, and gray literature were not systematically searched. Titles and abstracts were screened for relevance, with emphasis on prospective or randomized studies informing steroid dose, route, schedule, histologic response, toxicity, or biological plausibility. Because the aim was to integrate historical dose studies, contemporary clinical practice, and mechanistic literature within one clinically oriented review, the evidence was synthesized narratively rather than through formal systematic-review procedures.

Supplementary Table S1. Practice heterogeneity in glucocorticoid pulse therapy for TCMR.

| Practice Element | Typical Pattern | Main Implication |
| --- | --- | --- |
| Initial pulse dose | 250 mg, 500 mg, or 1,000 mg methylprednisolone | Considerable inter-center variation despite limited comparative evidence |
| Pulse duration | Usually 3 days, sometimes 5 days | Duration often reflects habit rather than trial-based optimization |
| Route | Predominantly intravenous; oral regimens rarely used | IV administration is conventional but not clearly proven superior in all settings |
| Lesions treated | Borderline, Banff IA/IB, some IIA episodes | Threshold for treatment varies, especially for borderline changes |
| Oral taper | Variable duration and intensity; no standardization | Cumulative GC exposure from taper may exceed the IV pulse component |
| Escalation strategy | ATG for steroid-resistant or higher-grade rejection | Criteria for steroid failure vary across centers |
| Response assessment | Creatinine trend, repeat biopsy in selected centers | Functional response may misclassify histologic outcome |

Patterns summarized from KDIGO guidance, contemporary reviews, and European, Canadian, and US practice surveys [1–4, 11, 12, 23].

Supplementary Table S2. Mechanistic layers of glucocorticoid action relevant to steroid pulse therapy in TCMR.

| Mechanistic Layer | Representative Effect | Relevance to Rejection | Key References |
| --- | --- | --- | --- |
| Genomic GR signaling/ NF-kappaB antagonism | Nuclear GR-mediated transcriptional reprogramming | Broad suppression of inflammatory mediators and leukocyte activation | [24–28] |
| IL-2/IL-2R/JAK-STAT suppression | Reduced STAT5 activation; impaired IL-2-dependent T-cell signaling | Directly limits alloreactive T-cell proliferation and survival | [38, 39] |
| Leukocyte trafficking | Redistribution of lymphocytes; reduced adhesion/tissue recruitment | Rapid reduction in intragraft inflammatory cell burden | [24, 32] |
| Rapid non-genomic effects | Membrane-associated and signaling-complex effects at high exposure | Explains fast onset of pulse therapy | [32–35] |
| Immunometabolic reprogramming | Reduced glycolytic switch; altered mitochondrial/TCA-cycle programs | Pushes immune cells away from inflammatory effector states | [40–42] |

*GR = glucocorticoid receptor; TCA = tricarboxylic acid.*

Supplementary Table S3. Comparative pharmacological properties of clinically used glucocorticoids.

| Agent | GC potency | MC potency | Equiv. dose (mg) | Biol. t1/2 (h) | Plasma t1/2 (h) | Oral bioavail. (%) |
| --- | --- | --- | --- | --- | --- | --- |
| Hydrocortisone | 1 | 1 | 20 | 8–12 | 1.5–2.0 | ~96 |
| Prednisone* | 4 | 0.8 | 5 | 12–36 | 2.1–3.5 | ~80 |
| Prednisolone | 4 | 0.8 | 5 | 12–36 | 2.1–3.5 | 82–99 |
| Methylprednisolone | 5 | 0.5 | 4 | 12–36 | 2.3–4.0 | 82–89 |
| Dexamethasone | 25–30 | ~0 | 0.75 | 36–72 | 3.0–4.5 | 78–81 |

*GC = glucocorticoid; MC = mineralocorticoid; t1/2 = half-life. Prednisone is a prodrug; values refer to active metabolite prednisolone. Data compiled from Czock et al. [46], Bergmann et al. [47].**

Supplementary Table S4. Banff classification of TCMR.

| CATEGORY 4: TCMR  *Acute TCMR IA* Banff Lesion Score i ≥ 2 AND Banff Lesion Score t2  *Acute TCMR IB* Banff Lesion Score i ≥ 2 AND Banff Lesion Score t3  *Acute TCMR IIA* Banff Lesion Score v1 regardless of Banff Lesion Scores i or t  *Acute TCMR IIB* Banff Lesion Score v2 regardless of Banff Lesion Scores i or t  *Acute TCMR III* Banff Lesion Score v3 regardless of Banff Lesion Scores i or t  *Chronic Active TCMR Grade IA* Banff Lesion Score ti ≥ 2 AND Banff Lesion Score i-IFTA ≥ 2, other known causes of i-IFTA (eg, pyelonephritis, BK-virus nephritis etc.) ruled out AND Banff Lesion Score t2  *Chronic Active TCMR Grade IB* Banff Lesion Score ti ≥ 2 AND Banff Lesion Score i-IFTA ≥ 2, other known causes of i-IFTA ruled out AND Banff Lesion Score t3  *Chronic Active TCMR Grade II* Arterial intimal fibrosis with mononuclear cell inflammation in fibrosis and formation of neointima |
| --- |
|  |
